# International comparison of experience-based health state values at the population level

**DOI:** 10.1186/s12955-017-0694-9

**Published:** 2017-07-07

**Authors:** Richard Heijink, Peter Reitmeir, Reiner Leidl

**Affiliations:** 1 National Institute for Public Health and the Environment, P.O. Box 1, 3720, BA Bilthoven, the Netherlands; 2 Dutch Healthcare Authority, Utrecht, the Netherlands; 3 Institute for Health Economic and Healthcare Management, Helmholtz Zentrum München, Neuherberg, Germany; 4 Munich Center of Health Sciences, Ludwig-Maximilians-University, Munich, Germany

## Abstract

**Background:**

Decision makers need to know whether health state values, an important component of summary measures of health, are valid for their target population. A key outcome is the individuals’ valuation of their current health. This experience-based perspective is increasingly used to derive health state values. This study is the first to compare such experience-based valuations at the population level across countries.

**Methods:**

We examined the relationship between respondents’ self-rated health as measured by the EQ-VAS, and the different dimensions and levels of the EQ-5D-3 L. The dataset included almost 32,000 survey respondents from 15 countries. We estimated generalized linear models with logit link function, including country-specific models and pooled-data models with country effects.

**Results:**

The results showed significant and meaningful differences in the valuation of health states and individual health dimensions between countries, even though similarities were present too. Between countries, coefficients correlated positively for the values of mobility, self-care and usual activities, but not for the values of pain and anxiety, thus underlining structural differences.

**Conclusions:**

The findings indicate that, ideally, population-specific experience-based value sets are developed and used for the calculation of health outcomes. Otherwise, sensitivity analyses are needed. Furthermore, transferring the results of foreign studies into the national context should be performed with caution. We recommend future studies to investigate the causes of differences in experience-based health state values through a single international study possibly complemented with qualitative research on the determinants of valuation.

**Electronic supplementary material:**

The online version of this article (doi:10.1186/s12955-017-0694-9) contains supplementary material, which is available to authorized users.

## Background

Health state values are used to weigh the different dimensions of a particular health state, such as physical functioning and mental health.^1^ By rendering the overall aggregation of health they make up a key methodological step in the evaluation of health effects. The concepts and methods used to generate health state values have been studied and discussed continuously, mainly considering the following questions: which health dimensions should be valued, how to elicit these values, and whose values should be used [1–3]? It has been shown that such conceptual and methodological choices affect outcomes at the population level as well as for patient groups defined by diseases [4–7]. Consequently, it is important for health care decision makers to be aware of the choices underlying the value sets they base their decisions on. A recent comparative study on diabetes patients, for example, concluded that “the choice of tariff might have substantial impact on funding decisions” [8].

Health state values have been elicited using choice-based experiments in which respondents are asked to make trade-offs between living in a less than perfect health state and living in full health.^2^ However, concerns have been raised regarding this approach. It requires respondents to assess hypothetical health states (HHS) and, in that case, healthy respondents may focus on the health problem they are asked to imagine, overlooking other health domains and underestimating adaptation. At the same, patients may have adapted to their health problems and be unable to predict their valuation of (or recall how they valued) being in full health [3, 9]. It has been argued that a different approach is needed which reflects the degree to which health states actually affect people, instead of respondents’ choices or ex-ante preferences regarding different hypothetical health states [3]. In recent years, several population-based studies used such an experience-based approach to elicit health state values for Germany, the U.S.A., Sweden and China [10–13]. The experience-based approach involves a generic rating by individuals on how they feel at a particular moment, complemented with concurrent descriptive information about their health status.^3^ Leidl et al. were the first to estimate a value set based on experienced health states (EHS) in Germany, relating respondents’ EQ-VAS rating of their own health (on a 0,100 scale) to their health status as described by the health dimensions of the EQ-5D-3 L [10]. The results indicated that such EHS-based valuations can differ from valuations of HHS.

To the best of our knowledge, EHS-based value sets were derived on a national basis only. A concern at this stage is the still limited number of countries for which EHS-based value sets are available. As a consequence, researchers in other countries could decide to use these (foreign) EHS-based value sets in their national-level analyses, similar to several studies that were based on HHS-based value sets [14–16]. However, as valuations may differ between countries, so may economic evaluations and population health assessments based upon them. Therefore, the transferability of these studies and their usefulness for national-level policy making depends on the cross-country comparability of health preferences. It can be argued that value sets should represent national preferences since reimbursement decisions mostly use a national perspective. More generally, health systems may be expected to *produce* health outcomes in accordance with the preferences of the population they serve and whose means are put in use.

From a theoretical point of view, it may be expected that health state valuations differ between countries [17–19]. Economic and geographical circumstances and social support systems vary between countries, affecting the way people perceive and value health limitations. In addition, the valuation of health states may be influenced by culturally or religiously defined preferences related to health. At least, this was found in some empirical studies on HHS-based value sets [15, 20–27]. In general, these studies concluded that cross-country variation in health state values cannot be ignored, even though the magnitude of the differences varied between studies and valuation methods. For example, Badia et al. found statistically significant differences between Spanish and UK respondents for 35% of the health states valued [20]. Spanish respondents placed significantly greater value on the functional dimensions mobility and self-care and lower value on pain and anxiety, compared to British respondents. Similarly, Norman et al. showed that mobility problems were considered more important among Japanese respondents compared to respondents from the UK, whereas opposite results were found for pain and anxiety [25]. An important limitation of cross-country comparisons of HHS-based value sets is the methodological variation between studies regarding e.g. the number and choice of health states valued by respondents and the algorithm used to establish the value set. For example, it was shown that methodological variation in the transformation of negative values explained a substantial part of the difference between the UK and US versions of HHS-based value sets for the EQ-5D-3 L value sets [28].

In this study, we aimed to expand the evidence on differences in health state values between countries. The study is the first to compare EHS-based valuations across countries. We analyse data from EQ-5D-3 L population surveys conducted in fifteen countries between 1993 and 2002. Similar to previous national studies [10, 29], we investigate the relationship between respondents’ EQ-VAS rating of their own health (outcome variable) and their descriptive health profile using the EQ-5D-3 L. The current study provides better generalizability of the results in comparison to previous international comparisons of health state values, by including 15 countries. Furthermore, using EHS-based valuations reduces methodological variation in terms of transformation and estimation procedures, compared to previous comparisons of HHS-based value sets. We focus on two research questions: (1) Does the mean observed EQ-VAS per health state (i.e., a combination of health dimensions) differ between countries? (2) Does the estimated value of particular health dimensions vary across populations, both in terms of the size of their impact and the ranking of dimensions? Results of the first part are expected to show the extent to which EHS-based valuations of particular health states vary across countries. Results of the second part are expected to demonstrate to what extent individual health dimensions are valued differently.

## Methods

### Data

Data was provided by the EuroQol Group, covering fifteen countries in which EQ-5D-3 L population surveys were conducted. The EQ-5D-3 L surveys were carried out between 1993 and 2002. All surveys used a standardized version of the EQ-5D-3 L, including the EQ-VAS and the EQ-5D-3 L descriptive profile. The translation process of the EQ-5D-3 L surveys followed the guidelines proposed in the international literature [30]. Survey respondents were non-institutionalized persons aged 18 years and older. Sample sizes varied between 400 and 5500 observations per country; in countries with more than one sample, samples were aggregated. In total 31,852 observations were included in the dataset. Additional file 1 provides more details about the original studies.

### EQ-VAS and EQ-5D-3 L descriptive profile

The outcome variable was the respondents’ rating of their own health at time of the interview using the EQ-VAS (0–100 scale ranging from the worst to the best imaginable health state). The main explanatory variables were the five dimensions covered in the EQ-5D-3 L descriptive health profile: mobility, self-care, usual activities, pain/discomfort, and anxiety/depression. Each respondent indicated whether he/she had “no problems”, “some problems” or “severe problems” in each of the five dimensions thus classifying into one of 243 possible health states. In most surveys, respondents also provided additional information about their age, gender, and education-level.^4^ Table 1 provides descriptive information about the samples in the pooled dataset.

**Table 1:** Descriptive information about the fifteen country samples in the dataset^a^

	ARM	BEL	CAN	FIN	GER	GRE	HUN	JAP	NET	NZL	SLV	SPA	SWE	UK	US
N	2222	1274	1518	2411	828	464	5503	620	1155	1328	742	2741	3603	3395	4048
Year of study	2002	2001	1997	1992	1994–1998	1998	2000	1998	1991–2003	1999	2000	1995–2000	1994–1998	1993	2002
Mean age	46.4	50.0	53.5	51.4	51.0	42.2	46.6	48.5	50.9	49.9	44.2	46.8	48.4	47.9	43.0
% Female	70.2	52.7	33.5	51.8	41.3	45.9	55.2	57.3	53.4	56.7	56.3	52.9	46.0	56.7	58.2
Mean EQ-VAS	65.7	80.2	78.8	76.8	80.1	79.0	71.1	77.8	80.8	80.8	76.4	75.9	83.3	82.5	84.3
Mean EQ-VAS adjusted^b^	65.6	81.2	80.6	78.2	81.2	77.1	70.9	78.3	82.0	81.8	75.3	75.7	83.6	82.8	82.9
Mobility (%)															
Moderate problems	26.0	15.6	21.9	27.7	21.8	12.6	18.6	7.3	9.9	19.7	29.4	13.5	10.7	18.3	17.8
Severe problems	1.4	0.2	0.3	0.4	0.2	0.7	0.9	0.0	0.1	0.3	0.4	0.3	0.2	0.1	0.3
Self-care (%)															
Moderate problems	12.0	3.7	3.5	7.3	2.9	5.2	5.4	1.8	4.2	4.0	13.4	2.6	1.4	4.1	4.0
Severe problems	2.2	0.7	0.5	0.8	1.1	0.5	1.1	0.0	0.3	0.4	0.5	0.3	0.5	0.2	0.4
Usual activities (%)															
Moderate problems	26.1	15.6	16.7	20.9	14.0	10.2	12.2	4.7	15.1	20.7	31.3	10.1	6.2	14.2	13.7
Severe problems	4.0	1.3	2.4	2.7	1.5	0.2	2.6	0.5	2.8	0.8	1.6	1.0	1.8	2.1	1.6
Pain/discomfort (%)															
Moderate problems	51.8	43.4	40.7	43.8	37.6	14.5	35.8	18.4	34.6	38.7	44.9	25.9	41.3	29.2	34.7
Severe problems	13.3	2.4	2.9	2.1	4.5	2.3	3.4	1.6	1.7	2.1	2.3	3.7	3.0	3.8	4.1
Anxiety/depression (%)															
Moderate problems	42.0	20.3	27.7	13.7	18.6	8.3	31.5	7.7	16.5	20.5	35.0	14.6	27.5	19.1	23.6
Severe problems	11.4	1.1	0.9	0.9	0.7	2.4	3.7	0.8	1.2	0.8	1.5	1.9	1.5	1.8	2.7

^a^ *ARM* Armenia, *BEL* Belgium, *CAN* Canada, *FIN* Finland, *GER* Germany, *GRE* Greece, *HUN* Hungary, *JAP* Japan, *NET* Netherlands, *NZL* New Zealand, *SLV* Slovenia, *SPA* Spain, *SWE* Sweden, *UK* United Kingdom, *US* United States

^b^Adjusted for age and gender using OLS to calculate prediction

### Analysis

We investigated the association between the EQ-VAS and the EQ-5D-3 L descriptive health profile. Since we focused on EHS-based valuations, there is one observation for each respondent in the dataset, in contrast to HHS-based valuation studies in which respondents assess multiple health states.

Regarding the first research question, we explored the distribution of EQ-VAS ratings by health state as observed in the country samples. We investigated those health states for which reliable and detailed comparison could be made, based on frequency of occurrence. These health states varied in terms of severity, in one or more dimensions, and covered 70% of the population analysed. We employed nonparametric tests for ordinal data to compare the distribution of the EQ-VAS ratings for these health states across countries [31]. We used the Kruskal-Wallis test, which tests whether multiple samples are from the same population. In addition, we used the Mann-Whitney-U test (or Wilcoxon-rank-sum test) which tests whether two independent samples are from populations with the same distribution. The latter was used to test the distribution of EQ-VAS ratings country-by-country.

Regarding the second research question, we estimated the value of particular health dimensions using regression models in which EQ-VAS ratings were regressed on health dimensions and levels of the EQ-5D-3 L descriptive profile. As shown by Leidl et al., commonly used (generalized/ordinary) least squares regression models for these type of data, as in e.g. [32], do not account for two methodological issues: predictions falling outside the original EQ-VAS range and inconsistent coefficients (i.e. coefficients predicting a higher value for a health state with more problems compared to a health state with less problems). Leidl et al. found more consistent outcomes with similar or better predictive accuracy using: 1) a generalized linear model with a logit link function (assuming a binomial distribution for the dependent variable^5^); 2) a restriction for the coefficients to create all non-positive parameter estimates; and 3) an alternative specification of the explanatory variables. Based on the consistency assumption that increases in problems may not increase the valuation of a health state, we employed constraints to the possible parameter estimates in the regression model. In consequence, optimal regression results were simultaneously obtained for all parameters (which is not guaranteed when excluding single parameters from the model).

Two variables were created for each of the five EQ-5D-3 L dimensions: one dummy variable for having any (some or extreme) problems versus no problems (*Mobility*, *Selfcare*, *Activity*, *Pain* and *Anxiety*) and one dummy variable for having extreme problems versus no extreme (none or some) problems (*Mobility3*, *Selfcare3*, *Activity3*, *Pain3* and *Anxiety3*).^6^ In this way, consistency is obtained, if parameter estimates are constrained as non-positive. In order to take into account the substantial number of respondents who did not report any problems, two intercept terms were included: one for the group of respondents who do not incur problems in any dimension, and one for all others (*INT1* and *INT2*). The basic VAS-level for people who experienced at least one problem in one of the five dimensions may thus differ from that for people who did not experience any problem. Summarizing, twelve explanatory variables were included reflecting the different elements of the EQ-5D-3 L descriptive profile.

We applied this specification to our data and estimated 15 country-specific regression models to investigate the value of different health dimensions at the country level. Furthermore, we used the pooled dataset of all countries to test whether the value of specific health dimensions differed significantly from one country to another. For this pooled-data set, we estimated 12 regression models referring each to one type of explanatory variable: existence of problems in each of the five dimensions, existence of extreme problems respectively, and the two intercepts. In each model, we included all explanatory variables while allowing the referred variable to vary by country using interaction terms. For example, we estimated one model in which we tested whether the impact of some or extreme mobility problems varied across countries. This model included all twelve explanatory variables plus interaction terms between country dummies and the health dimension mobility (having some or extreme problems versus no problems). In all pooled data models, random intercepts (*INT1* and *INT2*) were used. Using likelihood ratio tests, we then examined whether these models with interaction terms were statistically significantly different from models without interaction terms.

Finally, as a sensitivity analysis, we tested whether certain survey and respondent characteristics could further explain the variation in EQ-VAS ratings, beyond the different health dimensions and country effects. Previous studies showed that the data collection mode and respondent characteristics as age and gender explained part of the variation in health state values. Therefore, we added a dummy variable reflecting the data collection mode (postal survey or face-to-face interview), and respondent characteristics age and gender to the regression model. The computations for all regression models were performed using the NLMIXED procedure in SAS.

## Results

### Mean observed EQ-VAS per country for different health states

**Fig. 1:**
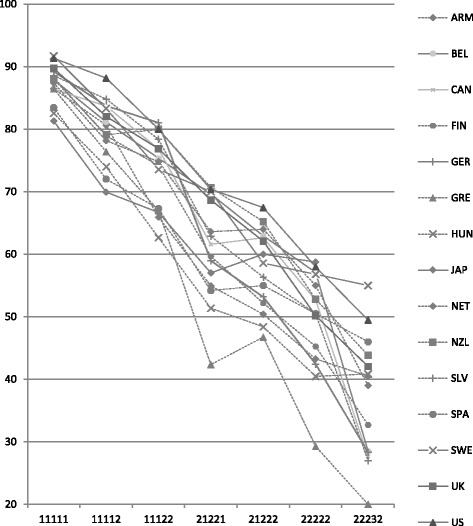
Mean EQ-VAS by health state and country. Seven frequently occurring health states were selected (see text). ARM = Armenia, BEL = Belgium, CAN = Canada, FIN = Finland, GER = Germany, GRE = Greece, HUN = Hungary, JAP = Japan, NET = Netherlands, NZL = New Zealand, SLV = Slovenia, SPA = Spain, SWE = Sweden, UK = United Kingdom, US = United States

Figure 1 shows that the mean EQ-VAS per health state varied between countries. For example, it ranged between 81.3 (Japan) and 91.7 (Sweden) for health state 11,111 (no problems in all dimensions); between 62.7 (Hungary) and 81.0 (Germany) for health state 11,122 (some problems in the dimensions pain and anxiety); and between 46.8 (Greece) and 67.5 (US) for health state 21,222 (some problems in all dimensions except self-care). For the first five health states in Fig. 1, the mean EQ-VAS differed on average 6.5 points (SD = 4.5) between countries. Differences between countries seemed greater for health states with more problems, but as the number of observations decrease with worse health, uncertainty is also increasing. The Kruskal-Wallis tests rejected the hypothesis that all samples were from the same population for all health states in Fig. 1 except for the worst health state (22232). Country-by-country comparisons using the Mann-Whitney-U test (statistics not shown here) demonstrated a similar pattern. These were less often significant for health states with more problems in the EQ-5D-3 L dimensions, even though the mean differences between countries were often greater. Countries at the low-end and high-end of the EQ-VAS scale differed from all other countries, in particular for health states including fewer problems. For example, Japan (lowest) and Sweden (highest) were significantly different from all other countries with regard to the value of health state 11,111. At the same time, Belgium, with a medium EQ-VAS rating for health state 11,111, differed from seven of the other countries in the dataset. For Japan, the distribution of EQ-VAS ratings also differed from six of the other countries for health state 11,122 but did not differ significantly from any of the countries for health state 22,222. For the more healthy states, the mean EQ-VAS was lowest in Hungary, Greece, Japan and Spain, and highest in the US, Germany, Slovenia, Sweden and the UK.

### Estimated valuation of individual health dimensions

Table 2 shows the results of the country-specific regression models. As the parameter estimates were forced to be non-positive, coefficients with a zero value indicate that the best estimate is found on this boundary. For most countries, having some or extreme problems with mobility, self-care, usual activities, pain, and anxiety had a statistically significant impact on the EQ-VAS rating (columns Mobility-Anxiety). For the additional effect of extreme problems, estimates were more often at the boundary (zero) and less often statistically significant (variables Mobility3-Anxiety3). In particular, the additional effect of extreme problems in mobility or self-care was not significant in most cases, whereas the additional impact of extreme problems regarding pain or anxiety/depression was almost always significant. Having *some or extreme problems* in the pain/discomfort dimension (for Sweden, Armenia and Hungary) or the usual activities dimension (for all other countries) showed a greater impact compared to the dimensions mobility, self-care and anxiety. The latter two showed the smallest effect on EQ-VAS ratings. There was much greater variation in the ranking of dimensions when respondents had *extreme problems*. Table 2 also underlines that the size of the value loss associated with each dimension differed between countries. The model parameters shown in Table 2 can be transformed into an EQ-VAS rating.^7^ For example, the mean EQ-VAS rating for health state 11,111 (no problems in all dimensions) was similar for Armenian and Greek respondents, i.e. 0.86. However, the EQ-VAS rating associated with health state 21,111 (using the sum of the coefficients INT2 and Mobility) differed substantially: 0.76 for Armenian respondents and 0.62 for Greek respondents. In other words, the impact of mobility problems was much greater in Greece compared to Armenia. As another example, the Finnish EQ-VAS rating associated with health state 11,211 (some or extreme problems performing usual activities and no problems in all other dimensions) equals 0.74 using the sum of the Finnish coefficients INT2 and Activity. If the value of usual activity problems would have been similar to the UK (−.424 instead of −.590), then health state 11,211 would be associated with an EQ-VAS rating of 0.77.

**Table 2:**
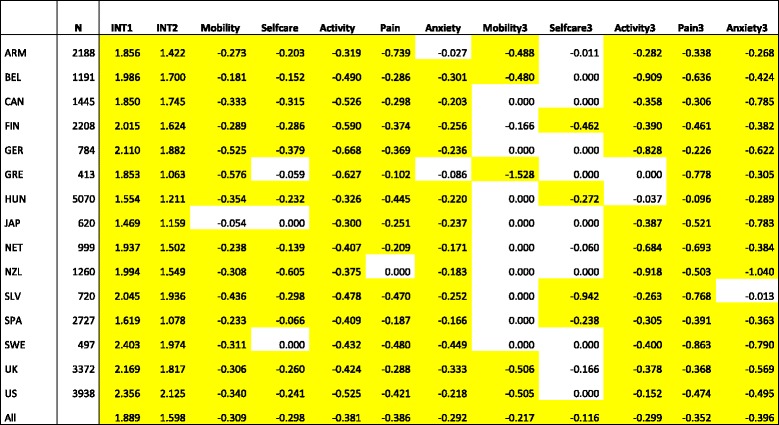
Coefficients country-specific regression models (yellow cells: *p* < 0.05)^a^

^a^ *ARM* Armenia, *BEL* Belgium, *CAN* Canada, *FIN* Finland, *GER* Germany, *GRE* Greece, *HUN* Hungary, *JAP* Japan, *NET* Netherlands, *NZL* New Zealand, *SLV* Slovenia, *SPA* Spain, *SWE* Sweden, *UK* United Kingdom, *US* United States

INT1 = Intercept - no problem in all dimensions; INT2 = Intercept – at least some problem in one dimension; Mobility = Some or extreme problems in Mobility; Selfcare = Some or extreme problems in Selfcare; Activity = Some or extreme problems in Activity; Pain = Some or extreme problems in Pain; Anxiety = Some or extreme problems in Anxiety; Mobility3 = Extreme problems in Mobility; Selfcare3 = Extreme problems in Selfcare; Activity3 = Extreme problems in Activity; Pain3 = Extreme problems in Pain; Anxiety3 = Extreme problems in Anxiety

**Fig. 2:**
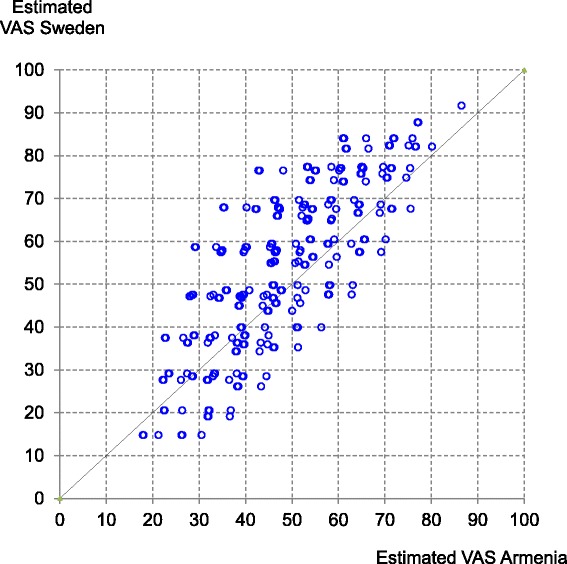
Estimated values for all 243 health states of the EQ-5D-3 L for Sweden and Armenia

To compare the values estimated for all possible combinations of health states, the countries with the highest (Sweden) and lowest (Armenia) observed mean EQ-VAS (Table 1) are taken as an example. Not for a single state, values were estimated the same for both countries: for 166 states, values in Sweden were higher while for 77 states, values were higher in Armenia (Fig. 2). The slope of the point cloud indicates equality of the overall structure of health state valuation while higher Swedish values relate to a significant, positive intercept of 5.4 points (regression results not shown).

Table 3 shows the results of the pooled data models. Each column represents a separate regression model in which one of the explanatory variables (the column header) was allowed to vary by country using interaction terms. Only the coefficients of these country-specific interaction terms are shown here, because they are the main parameters of interest. Overall, the models that included interaction terms at the level some or extreme problems (columns Mobility-Anxiety) were significantly different from models without these country-effects. The country-specific interaction terms showed significant differences (*p* < 0.01) at the level some or extreme problems for all countries, except for Japan in the dimensions mobility and self-care. For example, having some or extreme mobility problems was associated with greater value loss in Germany and Greece compared to all other countries. The impact of pain was largest in Armenia and Slovenia. The models with interaction terms for extreme problems (columns Mobility3-Anxiety3) were significantly different from models without interaction terms. At the same time, the country-specific interaction terms were less often statistically significant. We found no country-specific effect for severe mobility problems and self-care problems in Hungary, Spain, Canada and the Netherlands. The table also shows a particular association between the intercept term and interaction terms for some countries. For example, for Japan both the intercept term and the coefficients for mobility, self-care and activity were relatively low. In other words, Japanese respondents reported a relatively low EQ-VAS for health state 11,111 (no problems in all dimensions) in combination with the smallest value losses for problems with mobility, self-care and usual activities. In contrast, German respondents demonstrated the strongest effects in these three dimensions. Furthermore, Japan and four more countries (ARM, NET, SPA and SWE) showed a similar pattern in weak effects compared to GER, CAN, GRE and SLV with an opposite pattern of rather strong value decreases having such problems.

**Table 3:**
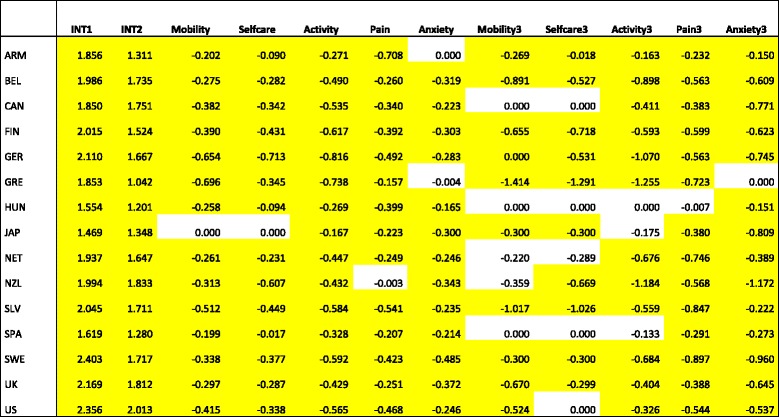
Interaction term coefficients for pooled-data regression models (yellow cells: *p* < 0.05)^a^

^a^ *ARM* Armenia, *BEL* Belgium, *CAN* Canada, *FIN* Finland, *GER* Germany, *GRE* Greece, *HUN* Hungary, *JAP* Japan, *NET* Netherlands, *NZL* New Zealand, *SLV* Slovenia, *SPA* Spain, *SWE* Sweden, *UK* United Kingdom, *US* United States

INT1 = Intercept - no problem in all dimensions; INT2 = Intercept – at least some problem in one dimension; Mobility = Some or extreme problems in Mobility; Selfcare = Some or extreme problems in Selfcare; Activity = Some or extreme problems in Activity; Pain = Some or extreme problems in Pain; Anxiety = Some or extreme problems in Anxiety; Mobility3 = Extreme problems in Mobility; Selfcare3 = Extreme problems in Selfcare; Activity3 = Extreme problems in Activity; Pain3 = Extreme problems in Pain; Anxiety3 = Extreme problems in Anxiety

Table 4 shows the ranking of countries for each model based on these coefficients. A high rank is equal to a relatively low value for a country in a particular dimension. A strongly positive correlation appeared between the interaction terms for mobility, self-care and usual activities (Spearman rank correlation between 0.8 and 0.9). Populations from countries with a relatively high value for mobility problems thus generally also attributed a high value to problems with self-care and usual activities. At the same time, there was little correlation between the interaction terms for pain and anxiety and those for the other dimensions.

**Table 4:** Ranking of countries (1-country with lowest value; 11-country with highest value) according to the interaction term coefficients^a^

	INT1	INT2	Mobility	Selfcare	Activity	Pain	Anxiety	Mobility3	Selfcare3	Activity3	Pain3	Anxiety3
ARM	10	11	5	7	2	15	1	12	9	5	4	2
BEL	8	7	2	6	10	6	13	11	1	14	11	8
CAN	12	6	10	13	12	8	6	1	1	7	3	13
FIN	6	8	6	11	13	10	12	10	14	10	7	6
GER	4	4	14	14	15	9	9	1	1	13	2	11
GRE	11	15	15	3	14	2	2	15	1	1	14	4
HUN	14	12	12	8	3	12	8	1	13	2	1	3
JAP	15	13	1	1	1	5	10	1	1	9	10	12
NET	9	10	4	5	5	4	4	1	10	12	12	7
NZL	7	9	8	15	4	1	5	1	1	15	9	15
SLV	5	3	13	12	9	13	11	1	15	4	13	1
SPA	13	14	3	4	6	3	3	1	12	6	6	5
SWE	1	2	9	1	8	14	15	1	1	11	15	14
UK	3	5	7	10	7	7	14	14	11	8	5	10
US	2	1	11	9	11	11	7	13	1	3	8	9

^a^ *ARM* Armenia, *BEL* Belgium, *CAN* Canada, *FIN* Finland, *GER* Germany, *GRE* Greece, *HUN* Hungary, *JAP* Japan, *NET* Netherlands, *NZL* New Zealand, *SLV* Slovenia, *SPA* Spain, *SWE* Sweden, *UK* United Kingdom, *US* United States

INT1 = Intercept - no problem in all dimensions; INT2 = Intercept – at least some problem in one dimension; Mobility = Some or extreme problems in Mobility; Selfcare = Some or extreme problems in Selfcare; Activity = Some or extreme problems in Activity; Pain = Some or extreme problems in Pain; Anxiety = Some or extreme problems in Anxiety; Mobility3 = Extreme problems in Mobility; Selfcare3 = Extreme problems in Selfcare; Activity3 = Extreme problems in Activity; Pain3 = Extreme problems in Pain; Anxiety3 = Extreme problems in Anxiety

**Fig. 3:**
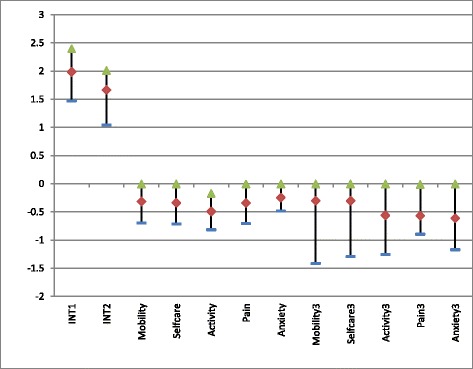
Range of the county-specific interaction term coefficients by EQ-5D-3 L health dimensions – maximum (*green*), median (*red*) and minimum (*blue*). As the parameter estimates were forced to be non-positive, coefficients with a zero value indicate that the best estimate is found on this boundary. Coefficients with zero value were found in almost all dimensions. INT1 = Intercept - no problem in all dimensions; INT2 = Intercept – at least some problem in one dimension; Mobility = Some or extreme problems in Mobility; Selfcare = Some or extreme problems in Selfcare; Activity = Some or extreme problems in Activity; Pain = Some or extreme problems in Pain; Anxiety = Some or extreme problems in Anxiety; Mobility3 = Extreme problems in Mobility; Selfcare3 = Extreme problems in Selfcare; Activity3 = Extreme problems in Activity; Pain3 = Extreme problems in Pain; Anxiety3 = Extreme problems in Anxiety

Figure 3 visualizes the range of the interaction term coefficients between countries for each model. It indicates that differences between countries were lower for health states with some or extreme problems than for those with extreme problems and for the intercept terms, the latter indicating the value of no problems.

Finally, in the sensitivity analysis, the data collection mode and the respondent characteristics age and gender were found to have a statistically significant impact in all models (results not shown here). On average, the inclusion of these variables reduced the country-specific interaction term coefficients, even though in some cases opposite results were found. Differences between countries changed to some extent, though the correlation between the interaction term coefficients before and after this adjustment was greater than 0.9 for 8 out of 12 models.

## Discussion

In this study, we investigated EHS-based valuations from a population perspective, using pooled data from 15 countries and standardized, novel methods. The study is the first to compare valuations based on experienced health states across countries. Previous international studies focused on cross-country differences in HHS-based value sets (see e.g. [7, 21]). We first studied the mean EQ-VAS ratings associated with particular health states and found that these varied between countries. Differences were most evident for health states with fewer problems and for countries at the low-end and high-end on the EQ-VAS scale. Next, we studied the impact of the individual health dimensions and found that different populations appear to rank the dimensions and problem levels of the EQ-5D-3 L in different ways. For example, the impact of having some or extreme problems in the pain dimension was relatively high (compared to other dimensions) for Armenian and Swedish respondents, while it was low for respondents from New Zealand. In most countries, having some or extreme problems in the usual activities dimension had the largest impact, which shows that similarities between countries were present too. The magnitude of the health dimensions’ coefficients also varied between countries, which will translate into non-negligible differences in terms of health outcomes. As illustrated in the results section, the variation in coefficients may very well generate a 7-point difference on the EQ-VAS 0–100 scale, which was considered a minimally important difference from a clinical perspective in one study [33]. Estimations for all possible combinations of health dimensions also illustrated that systematic differences in terms of valuation may exist (Fig. 2). At the same time, even though individual health dimensions may be valued differently across countries, we did find a positive correlation between the valuation of mobility, self-care and usual activities. No correlation was found between the valuation of pain or anxiety and all other dimensions (a previous study found a similar pattern for Spanish respondents, see [20]). This indicates that the pain/discomfort and anxiety/depression dimensions represent different types of health problems compared to mobility, self-care and usual activities, which are valued differently as a result. At the level of extreme problems, differences between countries were less clear and more often not significant.

In spite of our standardized approach, we cannot exclude that methodological differences caused some of the variation between countries. The underlying population-surveys varied regarding the year in which the survey was conducted, the interview mode (postal or face-to-face interview), and the sampling procedure. In the regression models, interview mode was found to affect EQ-VAS ratings yet it did not change cross-country differences substantially, in particular regarding the relative importance of the different dimensions (see Additional file 2). Differences in study year may have affected the results, in case the VAS valuation of health changes over time due to e.g. changes in the health or social care system that affect valuation. To our best knowledge, there is no evidence on this issue regarding the samples used in this study, however. Differences in sampling procedures are described in Additional file 1. Not all studies reached the aim of including a representative sample of the underlying population, but differences in the distribution by age and gender across studies were taken into account in the regression model. After adjusting for differences in the distribution of these respondent characteristics and the interview mode, cross-country differences remained similar. Therefore, we argue that differences in health values between countries cannot be ignored. Interestingly, these differences may not necessarily reflect differences in the economic position of countries. Both wealthier and less-wealthier countries were found to value, for example, the reduction by some or extreme problems in mobility much heavier than other countries in the sample.

The existence of cross-country differences in EHS-based valuations resembles the results from previous studies on HHS-based value sets, yet our findings also differ in some respects. Remarkably, some or extreme problems with usual activities was associated with a large reduction of the EQ-VAS in all countries, whereas this dimension was much less important in most HHS-based value sets [24]. This may confirm the finding from Leidl et al.’s national studies that the two approaches may generate value sets with different characteristics at the population level. As argued in the introduction, EHS-based value sets can be used as an alternative for the traditional HHS-based value set. The EHS-based value set developed for Germany has, for example, been used to test the validity of the EQ-5D-3 L in specific patient groups [6, 34–37]. Recently, EHS-based value sets have also been used in clinical studies in Sweden, in line with the recommendation of the Swedish guidelines on economic evaluation to use the patient’s perspective [8, 38, 39]. In case the approach will be applied in an international setting, it becomes important to take cross-country differences in health values into account. For example, multinational clinical trials planning to use EHS-based values may not rely on a single value set from one country but should regard the need to adapt values to decision-specific contexts by using a respective value set, and to control for eventual sensitivity of results when basing evaluation on this country-specific valuation. In addition, our study confirms that researchers should be cautious when transferring health outcomes, based on EHS-based value sets, from foreign studies into national calculations. This may lead to invalid conclusions for their target population. Results also confirm that a simple adjustment formula does not seem to exist, because respondents in one country did not attach greater or smaller value to *all* dimensions. Rather, this pattern fluctuated between the different health dimensions and levels. Eventually, the findings warn decision makers both against using original EQ-VAS valuations alone without considering eventual adaptation to country context, as well as against the unreflected transfer of results derived from value sets of other countries.

The results must be interpreted with the following limitations in mind. The main methodological issues related to the EQ-VAS instrument are context bias, end-of-scale bias and response spreading [2, 40]. The use of the EQ-VAS to establish health state values has been criticised because of a perceived lack of theoretical foundation, yet Parkin and Devlin showed that it does have a theoretical foundation in (psychometric) measurement theory [40]. Context bias means that the value of a particular health state depends on which health state it is compared with. This relates to experiments in which respondents value multiple health states (HHS-based value set), yet in this study we used the single EQ-VAS rating associated with the current health state of the respondents. Dolan and Kahneman argued that the usefulness of EQ-VAS-type ratings also depends on any other comparisons respondents make at time of the assessment, e.g. between themselves and other people [9]. Based on our data we however could not assess whether this led to systematic cross-country variation and should be considered a measurement distortion. By focusing on respondents’ valuation of their current health state, this study could not consider the anchor point death as in HHS-based valuation studies. In HHS-based approaches, the value of death is commonly defined as zero and used as anchor point to adjust for differential response behaviour. There is also methodological discussion on how to further develop such anchoring in HHS-based valuation [41]. Previous population level studies with and without anchoring yet indicated that the difference may be limited [42]. For the examples of patients with heart disease and with inflammatory bowel disease, it has further been found that non-anchored VAS-based value sets correlate better or the same with accepted clinical measures as anchored, utility-based value sets [35, 37]. When calculating quality-adjusted survival in the experience-based approach, death is also zero because of zero survival time. Not attributing a value to death in the experience-based approach implies that negative valuations for health states do not exist, in contrast to traditional QALY calculations. Another point, end-of-scale bias refers to respondents avoiding the end-points of the EQ-VAS-scale. The latter may have affected our cross-country comparisons (the regression coefficients) if respondents in country A were more inclined to avoid end-points compared to respondents in country B. Although a substantial proportion of the respondents did report a EQ-VAS rating of (around) 100, the issue could not be tested with the data at hand.

In addition to these EQ-VAS-related issues, we should note that we did not include interaction terms between the different EQ-5D-3 L dimensions and levels. This would allow the effect of e.g. mobility to vary by different levels of self-care. However, previous studies on health valuation showed mixed results regarding model fit improvement after the inclusion of such interaction terms [24]. Moreover, adding multiplicative terms increases data requirements and makes interpretation of the model results much more complex. Another limitation was the limited sample size of some of the surveys. More importantly, the number of respondents with extreme problems in any or several dimensions was limited (the surveys did not include institutionalized persons and certain health problems may have hindered more severely ill people from participation). Therefore, there were relatively little EHS-based values for these dimensions, which reduced the precision of the estimates. In addition, it was unclear whether all types of respondents, according to the characteristics that may affect health valuation, were represented in the surveys. There is little evidence on the impact of respondent characteristics on health valuation though and we tested the impact of differences in distributions by age and sex. Finally, we applied a standardized methodology to an international data set provided by the EuroQol Group. For Sweden and Germany EHS-based value sets have been developed elsewhere using significantly larger and more recent samples [10, 11]. This should be considered before using respective data from this study. We cannot exclude that for some of the countries analyzed specification tests would suggest a different model-specification to be better. However, the main goal of *this* study was to compare the valuation of dimensions across countries. For reasons of comparability, we preferred using an identical regression framework for all samples.

## Conclusion

In sum, we explored international differences in EHS-based valuations. Decision makers, who want to focus on patient or individuals’ valuations when considering the effectiveness of medical interventions, may find EHS-based valuations a useful alternative to HHS-based valuations. When determining the effectiveness of a medical intervention, patients and physicians typically consider what has actually happened rather than preferences regarding what could occur. As one important consequence, the use of such EHS-based valuations has the potential to better integrate the assessment of medical effectiveness and cost-effectiveness. Overall, the results indicate that EHS-based valuations differ between countries, at least for the countries included in this study and for the dimensions and levels covered by the EQ-5D-3 L. This may depend upon different valuations of health problems, though systematic patterns explaining these differences between countries, or identification of clusters of countries similar in valuation remain to be recognized. Since health state values are an important input parameter in population health comparisons and evaluations of health interventions, these findings should be taken into account when nationally interpreting valuations from other countries, but also in international comparative studies. We recommend future research to focus on the causes of differences in health valuation through a single international study (at one point in time) possibly complemented with qualitative research on the determinants of health values.

## Additional files

Additional file 1: Survey characteristics. (DOCX 71 kb)

Additional file 2: Interaction term coefficients for pooled-data regression model, after adjustment for age, gender and survey method (yellow cells: *p* < 0.05). (DOCX 70 kb)

## Abbreviations

ARM: Armenia
BEL: Belgium
CAN: Canada
EHS: Experienced health states
EQ-5D-3 L: EuroQol
EQ-VAS: EuroQol visual analogue scale
FIN: Finland
GER: Germany
GRE: Greece
HHS: Hypothetical health states
HUN: Hungary
JAP: Japan
NET: Netherlands
NZL: New Zealand
QALY: Quality adjusted life years
SLV: Slovenia
SPA: Spain
SWE: Sweden
UK: United Kingdom
US: United States

## Footnotes

1 For example, the EQ-5D-3 L descriptive profile includes the following dimensions: mobility, self-care, usual activities, pain/discomfort, and anxiety/depression. Each dimension has three levels: severe problems, some problems, and no problems.

2 Most often, the Time Trade Off or the Standard Gamble technique is used. See e.g. Brazier et al. [9].

3 Dolan and Kahneman preferred ‘moment-to-moment measurements’ such as the day reconstruction method in which people are asked to rate on a single scale how they felt the day before [11]. However, the latter instruments have been applied to a limited extent and require further development, particularly in the area of health.

4 The education variable comprised three levels (low, medium, and high) based on two questions: “left school at minimum age?” & “having a degree or professional qualification?”. Yes&No = low education, No&No = medium eductation, No/Yes&Yes = high education. In a few countries, additional questions were used to identify the level of education.

5 As explained in Leidl et al.: *“The binomial distribution can be seen to reflect a (large) series of experiments in which a person with the true health state of* p *is being confronted with a number randomly drawn from the (0,1) range. This number is said to reflect a well-defined health state. The respondent is then asked whether or not his/her health state is at least as good as this health state. The share of experiments in which this person is expected to agree is p”* [33]

6 Previous HHS-based valuation studies used the following two variables for each dimension: a three-level ordinal variable (no, some, severe problems) and a dummy variable for severe problems versus no or some problems.

7 Transformation is based on the formula $\frac{ \exp (sum)}{1+ \exp (sum)}$, where sum equals the sum of the coefficients related to a particular health state.
