# Supplementary material for: International comparison of experience-based health state values at the population level
Source: Health Qual Life Outcomes. 2017 Jul 7;15:138. doi: 10.1186/s12955-017-0694-9 (PMC5501450; doi:10.1186/s12955-017-0694-9)
Supplement: Supplementary file 1 — Survey characteristics. (DOCX 71 kb) [file 12955_2017_694_MOESM1_ESM.docx]

## Additional file 1: Survey characteristics

| **Reference** | **Country** | **Reference year** | **Final sample** | **Other relevant characteristics** |
| --- | --- | --- | --- | --- |
| Gharagebakyan G. Ghukasyan H. Williams A. Szende A (2003). Social inequalities in self-reported health: Is Armenia different from Slovenia? 20th Plenary Meeting of the EuroQol Group. Discussion Papers: 79-87. | Armenia | 2002 | 2222 | -*Face to face interview* among all selected household members  -Random sample of households from five provinces  -All selected households participated (100%), yet not each member of the household (60%)  -Final sample ‘quite representative’ for Armenian population regarding age and sex |
| -- Cleemput I. Kind P. Kesteloot K (2004). Re-scaling social preference data: implications for modelling. *Eur J Health Econ* 49: 290-298.  -- Cleemput I (2010). A social preference valuations set for EQ-5D health states. *Eur J Health Econ* 11: 205-213. | Belgium | 2001 | 1274 | -*Postal survey* with one reminder  -Random sample from the Flemish population. Sexes evenly represented.  -50% response rate  - Final sample reflected general population in terms of sex and main activity (employment, student etc.) |
| Johnson JA. Pickard AS (2000). Comparison of the EQ-5D and SF-12 health surveys in a general population survey in Alberta. Canada. *Med Care* 38 (1): 115-21. | Canada | 1997 | 1518 | -*Postal survey* with no reminder  -Random sample from the province Alberta (Canada) from database with residential listings  -35% response rate  -Respondents were predominantly male, and employed in final sample |
| --Ohinmaa A. Sintonen H (1996). Modelling EuroQol values of Finnish adult population. EuroQol Plenary Meeting 1995. Discussion Papers: 161-172.  -- Ohinmaa A. Sintonen H (1999). Inconsistencies and modelling of the Finnish Euroqol (EQ-5D) preference values. EuroQol Plenary Meeting 1998. Discussion Papers: 161-172. | Finland | 1992 | 2411 | -*Postal survey* with two reminders  -Random sample from Finnish population using population register. Genders evenly represented  -65% response rate |
| Schulenburg J.-M. G. v. d. Claes C. Greiner W. Uber A (1996). The German version of the EuroQol quality of life questionnaire. EuroQol Plenary Meeting 1995. Discussion Papers: 135-161. | Germany  (1) | 1994 | 370 | *-Postal survey* with two reminders  -Random selection from German population using telephone register. Inclusion criterion in order to prevent bias from telephone-based selection.  -37%-56% response rate  -Final sample included too many 60+ years old and males |
| Claes C. Greiner W. Uber A. Schulenburg J-M vd. (1998) The new German version of the EuroQol quality of life questionnaire. Centre for Health Economics and Health System Research. Diskussionpaper Nr.10 | Germany (2) | 1997 | 121 | -*Postal survey* with no reminder  -Random selection from German population using telephone register. Inclusion criterion in order to prevent bias from telephone-based selection.  -16% response rate  -Final sample not fully representative of German population regarding age (60+ and males overrepresented) |
| --Claes C. Greiner W. Uber A. Schulenburg J-M. Graf v.d (1999). An interview-based comparison of the TTO and VAS given to EuroQol states of health by the general German population. EuroQol Plenary Meeting 1998. Discussion Papers: 13-39.  --Greiner W Claes C. Busschbach JJV. Schulenburg J-M vd Graf (2005). Validating the EQ-5D with time trade off for the German population. *Eur J Health Econ* 6(2):124-130. | Germany  (3) | 1997/1998 | 337 | -*Face to face interview* with 18 trained interviewers  -Random sample of addresses from telephone directory using zip code. All contacted by telephone to set-up face-to-face interview (via reply cards). Non-random selection for gender, because females were underrepresented in telephone directory.  -8.5% response rate (with 8.5% of those contacted by phone an appointment was made)  -females and aged 24-45 underrepresented in the sample compared to German population |
| Yfantopoulos Y (1999). Quality of life measurement and health production in Greece. EuroQol Plenary Meeting. Discussion Papers: 100-114. | Greece | 1998 | 464 | -*Face to face interview* with trained interviewers  -Quota sampling standardized for age and sex  -Final sample: age and sex distribution similar to Greek population |
| Szende A. Nemeth R. (2003). Health-related quality of life of the Hungarian population. *Orv Hetil* 144 (34): 1667-74. | Hungary | 2000 | 5503 | -*Self-administered interview*  -Part of the National Health Survey with representative sample of the population  -response rate unknown |
| Tsuchiya A. Ikeda S.. Ikegami N. Nishimura S. Sakai I. Fukuda T. Hamashima C. Hisashige A. Tamura M (2002). Estimating an EQ-5D population value set: the case of Japan. *Health Econ* 11 (4): 341-53. | Japan | 1998 | 620 | -*Face to face interview* with trained interviewers  -Two-stage (geographical units and individuals) random sampling using local registry of electorates in three regions  -65% response rate  -Age and sex does not represent local distribution in final sample, but: age and sex adjustment has little effect on results |
| Essink-Bot ML. Stouthard M. Bonsel GJ (1993). Generalizability of valuations on health states collected with the EuroQol questionnaire. *Health Economics* 2: 237-246. | Nether  lands(1) | 1991 | 857 | -*Postal survey* with two reminders  -Random selection of households in Rotterdam area based on postal code.  -62% response rate  -Final sample was not representative for Dutch population |
| Lamers L et al. (2006).The Dutch tariff: results and arguments for an effective design for national EQ-5D valuation studies*. Health Econ*. 15:1121-1132 | Nether  lands(2) | 2003 | 298 | *-Face to face interview* with trained interviewers  -Quota sampling to achieve representative sample from Dutch population regarding age and gender. Sampling from marketing research company’s respondent lists.  -Age and gender distribution corresponded with Dutch population |
| Devlin NJ. Hansen P. Kind P. Williams A (2003). Logical inconsistencies in survey respondents’ health state valuations – a methodological challenge for estimating social tariffs. *Health Econ.* 12:529-544. | New Zealand | 1999 | 1328 | -*Postal survey* with reminder  -Random sample of people on electoral roll which was ex ante conform age, sex and ethnic distribution  -50% response rate  -certain ethnic groups (Maori, Pacific Island groups) underrepresented as well as lower educated in final sample |
| Prevolnik Rupel V. Rebolj M (2001). The Slovenian VAS tariff based on valuations of EQ-5D health states from the general population. 17th Plenary Meeting of the EuroQol Group. Discussion Papers: 11-23. | Slovenia | 2000 | 742 | *-Postal survey* with no reminder  -Random sample from the Slovenian population  -24.4% response rate  -Final sample representative for the Slovenian population regarding age and sex |
| Gaminde I. Cabasés J (1996). Measuring valuations for health states among the general population in Navarra (Spain). 12^th^ EuroQol Plenary Meeting. Discussion Papers: 113-123. | Spain  (1) | 1995 | 300 | -*Self-administered interview* with assistance from trained interviewers  -Quota sampling (by age and sex) from Navarra region  -Sample representative regarding age and sex |
| Badia X. Roset M. Herdman M. Kind P (2001). A comparison of United Kingdom and Spanish general population time trade-off values for EQ-5D health states. *Medical Decision Making* 21 (1): 7-16. | Spain  (2) | 1996/1997 | 973 | -*Face to face interview* with 11 trained interviewers  *-*Quota sampling (by age, sex) from Barcelona region using primary health care database  -Final sample representative for the Spanish population regarding age and sex |
| Gaminde I. Roset M (2001). Quality adjusted life expectancy. 17th Plenary Meeting of the EuroQol Group. Discussion Papers: 173-183. | Spain  (3) | 1999/2000 | 1468 | *-Face to face interview*  -Random sample from Navarra region |
| Bjork S. Norinder A(1999). The weighting exercise for the Swedish version of the EuroQol. *Health Econ* 8 (2):117-26. | Sweden (1) | 1994 | 534 | -*Postal survey* with three reminders  -Random sample from a national address register  -53% response rate  -In final sample a slight overrepresentation of younger groups and men |
| Burström K. Johannesson M. Diderichsen F. Swedish population health-related quality of life results using the EQ-5D *Quality of Life Research* 10: 621-635. | Sweden (2) | 1998 | 3069 | -Postal survey with reminder by telephone  -Representative sample of Stockholm County population  -63% response rate  -Higher non-response in lower age groups |
| --Kind P. Dolan P. Gudex C. Williams A (1998). Variations in population health status: results from a United Kingdom national questionnaire survey *BMJ* 316 (7133): 736-41.  --Dolan P. Modeling valuations for EuroQol health states (1997). *Med Care* 35(11):1095-108. | UK | 1993 | 3395 | -*Face to face interview* with 92 trained interviewers  -Stratified random sample from national postcode address file with stratification by geographic and socioeconomic characteristics  -Final sample was representative of the noninstitutionalized UK population regarding age, sex and social class |
| Shaw JW. Johnson JA. Coons SJ (2005). US Valuation of the EQ-5D Health States Development and Testing of the D1 Valuation Model. *Med Care* 43: 203-220. | US | 2002 | 4048 | -*Face to face interview* with 110 interviewers  -Multistage probability sampling: sampling frame based on residential mailing lists, demographic data and oversampling of certain minority groups  -Oversampling of minority groups  -75% response rate  -Final sample representative |
