# Supplementary material for: International comparison of experience-based health state values at the population level
Source: Health Qual Life Outcomes. 2017 Jul 7;15:138. doi: 10.1186/s12955-017-0694-9 (PMC5501450; doi:10.1186/s12955-017-0694-9)
Supplement: Supplementary file 2 — Interaction term coefficients for pooled-data regression model, after adjustment for age, gender and survey method (yellow cells: p < 0.05). (DOCX 70 kb) [file 12955_2017_694_MOESM2_ESM.docx]

**Additional file 2: Interaction term coefficients for pooled-data regression model, after adjustment for age, gender and survey method (yellow cells: p<0.05)†**

|  | **INT1** | **INT2** | **Mobility** | **Selfcare** | **Activity** | **Pain** | **Anxiety** | **Mobility3** | **Selfcare3** | **Activity3** | **Pain3** | **Anxiety3** |
| --- | --- | --- | --- | --- | --- | --- | --- | --- | --- | --- | --- | --- |
| ARM | 1.850 | 1.351 | -0.149 | -0.035 | -0.254 | -0.680 | 0.000 | -0.250 | 0.000 | -0.177 | -0.233 | -0.103 |
| BEL | 2.310 | 2.052 | -0.190 | -0.228 | -0.499 | -0.273 | -0.342 | -0.335 | -0.415 | -0.977 | -0.599 | -0.665 |
| CAN | 2.202 | 2.093 | -0.302 | -0.278 | -0.521 | -0.329 | -0.249 | -0.231 | 0.000 | -0.464 | -0.463 | -0.909 |
| FIN | 2.320 | 1.854 | -0.293 | -0.391 | -0.576 | -0.378 | -0.359 | -0.348 | -0.719 | -0.634 | -0.722 | -0.730 |
| GER | 2.341 | 1.901 | -0.620 | -0.557 | -0.767 | -0.391 | -0.278 | -0.288 | -0.530 | -0.885 | -0.676 | -0.471 |
| GRE | 2.150 | 1.304 | -0.489 | -0.315 | -0.478 | -0.237 | -0.207 | -0.348 | -0.427 | -0.351 | -0.446 | -0.037 |
| HUN | 1.604 | 1.265 | -0.174 | -0.060 | -0.236 | -0.361 | -0.195 | 0.000 | 0.000 | 0.000 | -0.002 | -0.157 |
| JAP | 1.560 | 1.425 | -0.047 | -0.119 | -0.216 | -0.274 | -0.312 | -0.300 | -0.300 | -0.241 | -0.391 | -0.545 |
| NET | 2.197 | 1.905 | -0.238 | -0.175 | -0.391 | -0.251 | -0.267 | -0.295 | -0.259 | -0.676 | -0.598 | -0.348 |
| NZL | 2.312 | 2.145 | -0.238 | -0.616 | -0.423 | -0.033 | -0.365 | -0.300 | -0.445 | -0.949 | -0.571 | -1.016 |
| SLV | 2.322 | 1.989 | -0.432 | -0.438 | -0.572 | -0.452 | -0.254 | -0.336 | -0.556 | -0.567 | -0.729 | -0.256 |
| SPA | 1.689 | 1.347 | -0.112 | 0.000 | -0.313 | -0.195 | -0.244 | -0.232 | 0.000 | -0.053 | -0.259 | -0.225 |
| SWE | 2.692 | 2.013 | -0.289 | -0.318 | -0.468 | -0.341 | -0.448 | -0.300 | -0.300 | -0.680 | -0.574 | -0.858 |
| UK | 2.237 | 1.876 | -0.231 | -0.275 | -0.430 | -0.229 | -0.389 | -0.333 | -0.325 | -0.400 | -0.411 | -0.723 |
| US | 2.395 | 2.035 | -0.340 | -0.324 | -0.553 | -0.451 | -0.285 | -0.345 | 0.000 | -0.326 | -0.581 | -0.509 |

*†* ARM=Armenia, BEL=Belgium, CAN=Canada, FIN=Finland, GER=Germany, GRE=Greece, HUN=Hungary, JAP=Japan, NET=Netherlands, NZL=New Zealand, SLV=Slovenia, SPA=Spain, SWE=Sweden, UK=United Kingdom, US=United States.

INT1=Intercept - no problem in all dimensions; INT2=Intercept – at least some problem in one dimension; Mobility=Some or extreme problems in Mobility; Selfcare= Some or extreme problems in Selfcare; Activity= Some or extreme problems in Activity; Pain=Some or extreme problems in Pain; Anxiety=Some or extreme problems in Anxiety; Mobility3=Extreme problems in Mobility; Selfcare3= Extreme problems in Selfcare; Activity3= Extreme problems in Activity; Pain3= Extreme problems in Pain; Anxiety3= Extreme problems in Anxiety.
